# Atomic‐Scale Mott–Schottky Heterojunctions of Boron Nitride Monolayer and Graphene as Metal‐Free Photocatalysts for Artificial Photosynthesis

**DOI:** 10.1002/advs.201800062

**Published:** 2018-05-15

**Authors:** Ke‐Xin Zhang, Hui Su, Hong‐Hui Wang, Jun‐Jun Zhang, Shu‐Yu Zhao, Weiwei Lei, Xiao Wei, Xin‐Hao Li, Jie‐Sheng Chen

**Affiliations:** ^1^ School of Chemistry and Chemical Engineering Shanghai Jiao Tong University Shanghai 200240 P. R. China; ^2^ Institute for Frontier Materials Deakin University Waurn Ponds Campus, 75 Pigdons Road Geelong Victoria 3216 Australia

**Keywords:** boron nitride, graphene, Mott–Schottky heterojunctions, photocatalysts

## Abstract

Heterojunction photocatalysts at present are still suffering from the low charge separation/transfer efficiency due to the poor charge mobility of semiconductor‐based photocatalysts. Atomic‐scale heterojunction‐type photocatalysts are regarded as a promising and effective strategy to overcome the drawbacks of traditional photocatalysts for higher photoenergy conversion efficiencies. Herein, an atomic‐scale heterojunction composed of a boron nitride monolayer and graphene (h‐BN‐C/G) is constructed to significantly shorten the charge transfer path to promote the activation of molecular oxygen for artificial photosynthesis (exemplified with oxidative coupling of amines to imines). As the thinnest heterojunction, h‐BN‐C/G gives the highest conversion, which is eightfold higher than that of the mechanical mixture of graphene and boron nitride monolayers. h‐BN‐C/G exhibits a high turnover frequency value (4.0 mmol benzylamine g^−1^ h^−1^), which is 2.5‐fold higher than that of the benchmark metal‐free photocatalyst in the literature under even critical conditions.

Efficiently utilizing renewable energy sources, especially solar energy, for photoenergy conversion has been regarded as a crucial measure to achieve the sustainable development.^1^, ^2^ Rational design of green photocatalysts was thus the key to realize this objective.^3^ Metal‐free photocatalysts are highly preferred due to their low cost, high abundance, and excellent stability for practical uses, despite great efforts in developing metal oxide, metal (oxy)sulfide, and metal (oxy)nitride photocatalysts and making full use of solar energy during the past decades.^4^, ^5^, ^6^, ^7^, ^8^, ^9^ Recently, a series of metal‐free photocatalysts from lightweight and abundant element, such as hexagonal carbon nitride (g‐C_3_N_4_), boron carbide, boron nitride, and nanocarbons, have emerged and drew intensive attention.^10^, ^11^, ^12^ These green photocatalysts have promised the possibility of constructing sustainable solar harvesting system for green chemistry, even though the catalytic efficiency of these photocatalysts are still needed to be further optimized.

Typical strategies to promote the activity of these metal‐free photocatalysts are mainly focused on elevating the crystallinity,^13^ engineering the mesoscale structure,^14^ and/or introducing dopants.^10^, ^15^ High crystallinity may enhance the charge diffusion both in the bulk and on the surface of a photocatalyst, elevating the final photoelectron conversion efficiency for certain redox reactions. Engineering the mesoscale structure can also improve the catalytic performance of a photocatalyst by shortening the diffusion path of reactants and also increasing the active surface area. Introducing dopants into the framework is more direct to tune the position of the valence band/or conduction band and thus finally regulate the redox strength of the metal‐free photocatalysts and also their selectivity for specific reactions. Recently, we introduced an alternative method to promote the catalytic activity of graphene/g‐C_3_N_4_ assemblies by constructing rectifying contact at their interface.^16^ The oxidation strength and the selectivity of the g‐C_3_N_4_ components were promoted via the Mott–Schottky effect at the interface of the graphene and g‐C_3_N_4_. Nevertheless, most metal‐free photocatalysts are still suffering from the poor mobility, as the intrinsic property of semiconductor‐based photocatalysts even engineered into nanostructures, and thus low activity for specific photocatalytic reactions, especially in the realm of artificial photosynthesis. As the thinnest heterojunction, the dyadic bilayers of graphene‐like 2D metal‐free materials can significantly shorten the charge transfer path to promote its activity for artificial photosynthesis, even though the synthesis of atomic‐scale heterojunction‐based photocatalyst still remain a great challenge for material scientists and chemists.

Thus, we turned toward the atomic‐scale heterojunction based on graphene and hexagonal boron nitride (h‐BN) monolayers for the construction of photocatalysts for highly efficient artificial photosynthesis. We here establish that highly selective and oxidative coupling reactions of amines, as a model reaction, using molecular oxygen as the green oxidant are efficiently catalyzed by the metal‐free photocatalyst of the atomic‐scale Mott–Schottky heterojunctions of boron nitride and graphene (h‐BN‐C/G). Albeit h‐BN with a wide bandgap is nearly inactive for visible‐light photocatalytic reactions, a little amount of carbon dopants introduced into the framework of h‐BN can reduce its bandgap largely and make it responsive to visible light. More importantly, the ultrathin structure of h‐BN‐C/G can principally enlarge the active surface area for possible catalytic reactions and also make the nanoheterojunction transparent for maximizing the utilization ratio of the light irradiation in a heterogeneous catalytic system.

Graphene and h‐BN have similar crystal structures with a lattice constant difference of only 2%,^17^ directly suggesting the great challenge in preparing heterojunctions of graphene and h‐BN rather than a ternary B‐C‐N alloy (BCN).^17^, ^18^, ^19^, ^20^ Pioneering work had shown the possibility of generating domains of h‐BN inside the graphene layers via introducing B, N‐rich precursors during the chemical vapor deposition process of graphene on a substrate.^21^ The bilayer heterojunctions composed of graphene and carbon‐doped h‐BN (h‐BN‐C) monolayer with promising physicochemical properties have never been analyzed as catalysts or photocatalysts till now due to the great challenge in material synthesis. Recently, graphene or few layers of both h‐BN and BCN have been synthesized via a nanoconfinement method from homogeneous mixture of B, N, and/or C‐rich molecules. It should be noted that such a homogeneous mixture of the precursors was all made by removing the solvent of a homogeneous solution.^22^, ^23^, ^24^ In this work, a modified nanoconfinement method was applied for the direct synthesis of the atomic‐scale heterojunctions of graphene and boron nitride, h‐BN‐C/G, by generating C‐rich domains and B‐rich domains in the solid precursor. The key step to generate separated C‐rich (gray in **Figure** **1**a) and B‐rich (blue in Figure 1a) domains in the precursor is to mix the anhydrous carbon source (glucose) and boron source (boric acid) with the leaving reagent (urea). Unlike the method used for preparing the homogeneous mixture of precursor molecules via removal of solvent from their solution, we directly mixed all the anhydrous raw materials by an agitator. The as‐obtained white powder was transferred to a covered crucible and heated to 1000 °C in a nitrogen oven. The as‐obtained powders were directly used for further characterizations. A yellow intermediate graphitic carbon nitride (g‐C_3_N_4_) acts as the lamellar templates for further growth of h‐BN and graphene inside their interlayers spaces. A high temperature (>750 °C) can lead to the thorough removal of g‐C_3_N_4_, releasing the confined BN and Gr layers simultaneously. The synthetic process was depicted in Figure 1a and Figure S1 (Supporting Information) with all detailed experimental parameters for typical h‐BN‐C/G and also control samples listed in Table S1 (Supporting Information). A series of h‐BN‐C/G‐*x* heterojunction composites were obtained by changing the weight percentage of glucose in boric acid (*x* = 0, 1.6, 15, 20) with fixed amounts of urea (Figure 1 and Table S1, Supporting Information).

**Figure 1:**
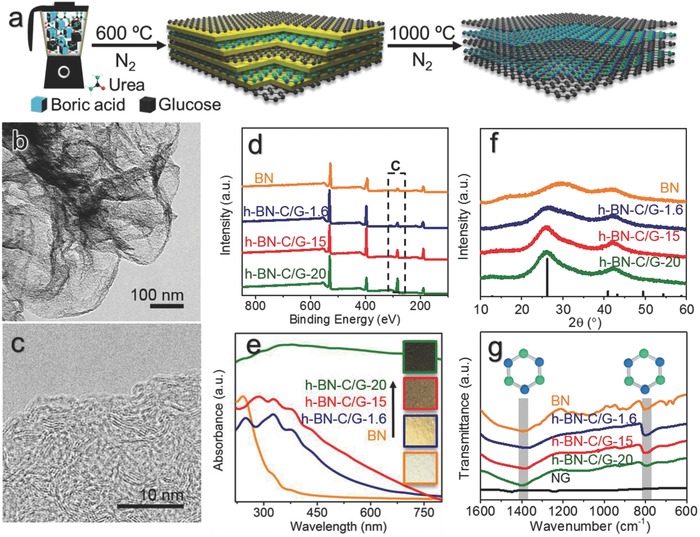
Synthesis and characterizations of h‐BN‐C/G‐*x* dyads. a) Proposed synthetic paths for preparing h‐BN‐C/G‐*x*. b) TEM and c) HRTEM images of the h‐BN‐C/G‐15 dyad. d) XPS, e) ultraviolet–visible absorption, f) powder XRD (PDF No. 73‐2095), and g) FTIR spectra of the h‐BN‐C/G‐*x* samples. The FTIR spectra (black line in (g)) of the pristine graphene sample obtained via similar method (NG) are also shown for comparison.

Large‐area scanning electron microscopy (SEM) (Figure S2, Supporting Information) and transmission electron microscopy (TEM) (Figure 1b, Figures S3 and S4, Supporting Information) images revealed that the as‐produced h‐BN‐C/G‐*x* composites are assemblies of continuous, flexible, and wrinkled sheets, similar to patched graphenes obviously developed in our group.^25^ The morphology of h‐BN‐C/G material was not obviously changed with varied weight ratios of glucose and boric acid and a fixed amount of urea. It should be noted that the BCN alloy nanosheets obtained from the homogeneous mixture of glucose and boric acid are holey nanosheets with a rich amount of through‐plane holes (Figure S5, Supporting Information). From the high‐resolution TEM (HRTEM) image (Figure 1c) of wrinkled part of the subunit nanosheets, multilayer structure of the h‐BN‐C/G sample was well observed with a lamellar stacking distance to be 0.33 nm.^26^, ^27^ X‐ray diffraction (XRD) patterns (Figure 1f) with weak and ultrabroad peaks also demonstrated the main components to be graphene and/or h‐BN few layers with typical peaks centered at 26° and 43° in all h‐BN‐C/G‐*x* samples and further excluded the formation of highly condensed graphite, cubic boron nitride, and other crystalline impurities. The estimated stacking distances of the h‐BN‐C/G sample from the XRD results were around 0.34 nm, slightly greater than that of the pristine BN sample (0.31 nm). The X‐ray photoemission spectroscopy (XPS) analysis results (Figure 1d) not only revealed the metal‐free feature of the h‐BN‐C/G‐*x* samples, but also confirmed the linear relationship between the final carbon contents and the amount of glucose added. The N 1s XPS peak (Figure S6, Supporting Information) demonstrates the chemical structure of N atoms mainly in the form of “pyridinic” N atoms and N—B bonds. The Fourier transform infrared spectroscopy (FTIR) (Figure 1g) further confirmed the existence of B—N bonds centered at 1380 and 780 cm^−1^ 18 with negligible amounts of C—N or C—B bonds, rather suggesting that only minor amounts of carbon atoms were introduced as heteroatoms into the lattice of h‐BN. The atomic ratios of boron and nitrogen element are nearly 1:1 for all h‐BN‐C/G‐*x* samples (Table S1, Supporting Information) with slightly more B atoms. Typical FTIR signal of cubic carbon nitride centered around 1200 cm^−1^ was not observed for all h‐BN‐C/G‐*x* samples. Indeed, the introduction of a slight amount of C dopants into the lattice of the h‐BN was well reflected by the obvious redshift in the optical absorption edge of h‐BN‐C/G‐1.6 from 245 nm for pristine BN as revealed by the UV–visible absorption spectra (Figure 1e) and also XPS analysis results (Figure S6, Supporting Information). The color of the h‐BN‐C/G‐*x* samples was thus changed from white to light yellow for pristine BN and h‐BN‐C/G‐1.6, respectively. However, further increasing the C contents in the h‐BN‐C/G‐*x* samples could obviously lead to the full absorption in the UV–visible absorption spectra, resulting in even darker samples (e.g., h‐BN‐C/G‐20). This observation directly demonstrated the formation of graphite domains in the samples and thus a possible Mott–Schottky heterojunction structure of BN (semiconductor) and graphene (semimetal).

We further analyzed the structural features of the h‐BN‐C/G catalyst to unveil the formation of nanoheterojunctions. TEM elemental mapping examination (**Figure** **2**a) directly revealed the heterojunction structure of C‐BN and graphene, packaged via a double‐layer structure, presumably due to the π–π* interaction between the two components with similar 2D conjugated network. The thickness of the nanoheterojunctions was directly demonstrated to be around 0.67 nm according to the atomic force microscope (AFM) analysis results (Figure 2a–e), rather speaking for the formation of an atomic‐scale heterojunction composed of only a single‐layer graphene and a boron nitride monolayer. Such a thin thickness of the 2D dyad with a super absorbing surface exhibited excellent dispersibility in water (Figure 2f). The aqueous colloidal solution of h‐BN‐C/G‐15 was highly transparent (Figure 2f), again due to the ultrathin structure of the 2D dyad. The high transmittance (Figure 2g) of the h‐BN‐C/G‐15 dispersion could facilitate the usages of light irradiation without significant loss caused by possible saturated absorption, resulting in photothermal conversion, and/or mirror reflection on the surface of a nontransparent solid photocatalyst.

**Figure 2:**
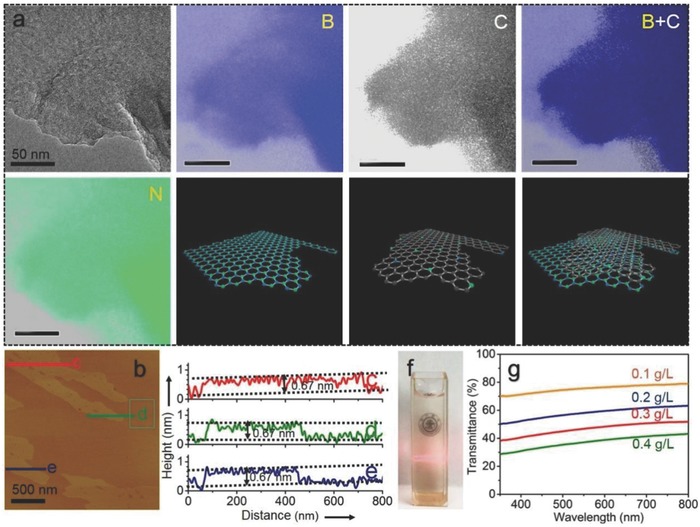
Structure and properties of the atomic‐scale heterojunction. a) TEM and elemental mapping images of the h‐BN‐C/G‐15 sample and corresponding schematic structures. b) AFM image of the h‐BN‐C/G‐15 sample and c–e) the height analysis results of selected parts. f) Photograph and g) the light transmittance spectra of the aqueous dispersion of the h‐BN‐C/G‐15 samples.

We initially chose the oxidative coupling reactions of amines to imines using molecular oxygen and visible light as a model reaction for evaluating the photocatalytic activity of the h‐BN‐C/G‐*x* and control samples in this work.^28^, ^29^ Coupling amines to imines is an important process in organic synthesis, the key step of which is to activate the molecular oxygen to assist the following dehydrogenation of amines and intermediates. A high reaction temperature has usually been required for metal‐free catalyst‐based reactions even under light irradiation to overcome the energy barrier of oxygen activation. Photocatalytic reactions were thus conducted at room temperature in acetonitrile as the optimized solvent (Table S2, Supporting Information) to compare the activity of h‐BN‐C/G‐*x*, BN, nitrogen‐doped graphene, and also benchmarked photocatalysts in the literature, including BCN alloy nanosheets (BNHG‐1000) and mesoporous carbon nitride (mpg‐C_3_N_4_). h‐BN‐C/G‐*x* materials and control samples showed excellent selectivity to the imines (**Table** **1** and Table S3, Supporting Information), but the conversion varied under fixed reaction conditions. The oxidative coupling reaction of benzylamine could not proceed in the absence of photocatalysts (Table 1, entry 1) light irradiation (Table 1, entry 8), or oxygen (Table 1, entry 9), indicating a photocatalytic process for the activation of oxygen molecules and the coupling reactions. Among all samples tested in this work, h‐BN‐C/G‐15 offered the best reactivity with a conversion of 95%, far surpassing the pristine BN (1.3%, Table 1, entry 4), h‐BN‐C/G‐20 (54.2%, Table 1, entry 7) with a higher carbon content, and also the benchmarked catalyst BNHG‐1000 (12.6%, Table 1, entry 2). These observations directly demonstrated the importance of the synergistic interactions between the BN and graphene components of dyadic h‐BN‐C/G‐*x* materials in tailoring the final catalytic activity. The mechanical mixture of BN and graphene layers (Table 1, entry 3) could only give a conversion of 11% under standard conditions, again speaking for the importance of the dyadic structure in promoting the catalytic activity.

**Table 1:**
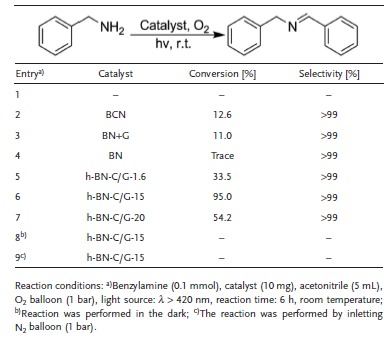
Study of reaction conditions

A further set of control reactions were tested to demonstrate the real role of h‐BN‐C/G‐15 as a photocatalyst in triggering the reaction. The high chemical stability of the h‐BN‐C/G‐15 catalyst was well reflected by the excellent reusability under standard conditions with nearly the same conversions in the following four runs (Figure S7, Supporting Information) and also unchanged structure (Figure S8, Supporting Information). Also, the final turnover number of the h‐BN‐C/G‐15 catalyst was estimated to be 1.82, calculated on the basis of conversions of the five successive recycling reactions, rather speaking for the role of the h‐BN‐C/G‐15 sample as a heterogeneous catalyst here. The dependence of conversions of benzylamine on the wavelength of light source matched well with the optical absorption of the h‐BN‐C/G‐15 material and thus demonstrated a photocatalytic process depending on its light absorption (**Figure** **3**a). A typical time course of benzylamine oxidation over the h‐BN‐C/G‐15 under standard conditions is shown in Figure 3b. The fact that the selectivity to imine product remained to be >99% for the whole oxidation process of benzylamine directly excluded possible overoxidation of benzylamine to benzyl aldehyde for further coupling reaction, as an alternative to the direct aminal intermediated path via deamination.^30^ To identify the generated reactive oxygen species (Figure 3c and Figure S9, Supporting Information), radical scavenger butylated hydroxytoluene (BHT), mannitol, catalase, and carotene were employed as scavengers for superoxide radical (O_2_^•−^), hydroxyl radical (•OH), hydrogen peroxide (H_2_O_2_), and singlet oxygen (^1^O_2_), respectively. The photocatalytic oxidation of benzylamine is obviously suppressed by the catalase, while negligible effects are observed when using other scavengers.^31^ As a result, a hydrogen peroxide‐assisted deamination photocatalytic process over the h‐BN‐C/G‐15 catalyst is proposed in Figure 3d.

**Figure 3:**
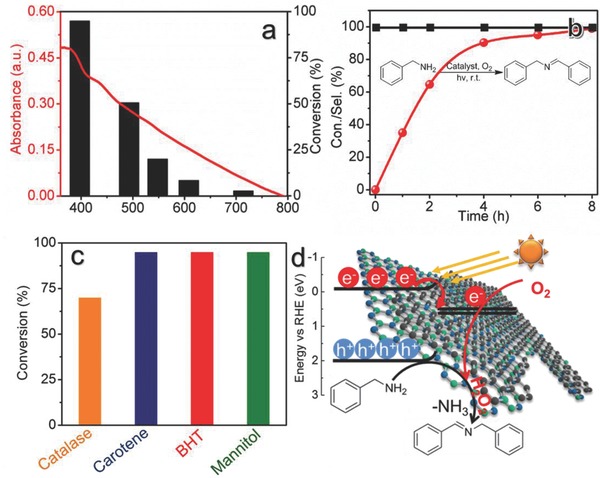
Photocatalytic activation of O_2_ over h‐BN‐C/G‐15 for selective oxidation. a) Wavelength‐dependent conversions of benzylamine by the h‐BN‐C/G‐15 photocatalyst. The ultraviolet–visible absorption spectrum of the h‐BN‐C/G‐15 catalyst is also presented as a reference. b) A time course of the conversions (red spheres) of benzylamine and corresponding selectivity to imine (black squares) by the h‐BN‐C/G‐15 photocatalyst using oxygen gas and visible light (λ > 420 nm). c) Conversions of benzylamine by the h‐BN‐C/G‐15 photocatalyst under standard conditions described in Table 1 except by adding inhibitors, including catalase, carotene, BHT, and mannitol. d) Proposed reaction mechanism over the h‐BN‐C/G‐15 photocatalyst via activating the oxygen gas to hydrogen peroxide for further oxidative coupling of benzylamine into imine.

The preadsorption and photocatalytic activation of benzylamine and oxygen molecules on the surface of graphene/h‐BN dyad were mainly induced by the electron relocalization and electron exchange under light irradiation, respectively, between the reactants and photocatalysts, largely depending on the band structure of the nanoheterojunctions. Considering the heterojunction structure of the h‐BN‐C/G‐*x* dyad with graphene as metal (semimetal) counterpart and boron nitride as semiconductor, optimizing the weight ratio of graphene and h‐BN was thus a direct way to modulate the interfacial Shottky barrier and thus the valance band position of the semiconductor part in the graphene/h‐BN dyad. The fixed photoluminescence peaks (**Figure** **4**a) of the h‐BN‐C/G‐*x* samples around 500 nm directly confirmed that the bandgap of the semiconductive h‐BN components in the h‐BN‐C/G‐*x* remained nearly the same with varied carbon contents. The interfacial electron redistribution of the Mott–Schottky heterojunction was monitored by the significantly and gradually decreased photoluminescence intensity of the h‐BN when more graphene domains were introduced (Figure 4a). Similar phenomenon was observed in the Raman spectra (Figure S10, Supporting Information) of h‐BN‐C/G‐40 samples with gradual decreased fluorescence interference with more graphene components introduced. Typical peaks for graphene at about 1587 (G band) and 1330 cm^−1^ (D band) were finally observed in the Raman spectrum of the h‐BN‐C/G‐40 sample after the fluorescence was completely quenched by the graphene components, again demonstrating the rectify contact between the h‐BN‐C and graphene components. Most importantly, the valance and conduction band position (Figure S11, Supporting Information) of the boron nitride part of h‐BN‐C/G‐15 sample were lowered obviously as compared with those of the h‐BN‐C/G‐1.6 sample presumably due to the band bending at the interface of Mott–Schottky heterojunction. This well explains the much higher catalytic activity of the h‐BN‐C/G‐15 catalyst with optimized weight ratio of graphene and h‐BN, as directly indicated by its remarkably high yield of the target product (95%), even though types of functional groups of all h‐BN‐C/G‐*x* catalysts are similar (Figure 1g). It should be noted that the normalized TOF values versus surface area (Figure S12 and Table S4, Supporting Information) also indicated an unchanged trend of the varied activities of all h‐BN‐C/G‐*x* samples, rather speaking for a minor contribution of the surface area to the final catalytic performance. As presented in Figure S12 (Supporting Information), the gradual decrease in the Brunauer–Emmett–Teller (BET) surface areas of BN, h‐BN‐C/G‐1.6, h‐BN‐C/G‐15, and h‐BN‐C/G‐20 samples was mainly induced by the formation of multilayer heterojunctions.

**Figure 4:**
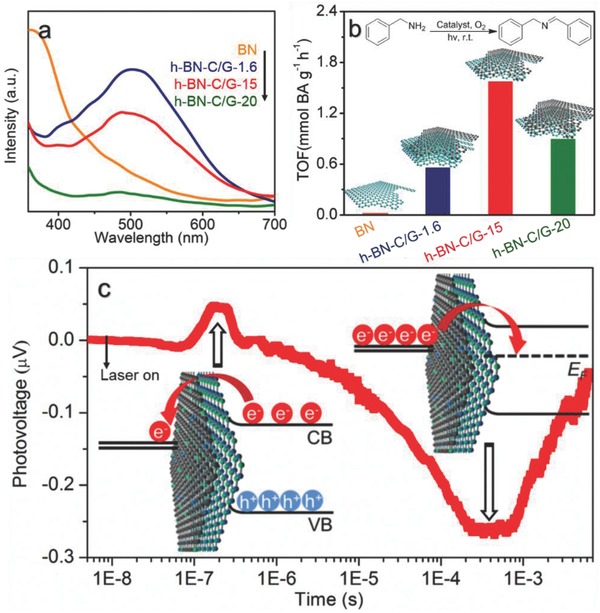
Mott–Schottky effect on the charge separation/transfer and the activities. a) Photoluminescence spectra and b) TOF values of the h‐BN‐C/G‐*x* samples and pristine BN. c) Transient photovoltage (TPV) spectrum of the best‐in‐class h‐BN‐C/G‐15 catalysts. The wavelength and intensity of excitation pulse laser are 355 nm and 125 µJ, respectively.

In order to investigate the dynamic properties of the atomic‐scale heterojunctions,^32^ the photovoltage transient (TPV) spectra of the h‐BN‐C/G‐15 sample (Figure 4c) were measured to deduce the moving direction of photogenerated charge carriers at the interface. Under the initiation of a pulse laser, the photogenerated electrons flow to the graphene side due to the difference between their work functions. Further interparticle diffusion of the separated electrons from the out surface to the inner part resulted in a positive TPV signal within a timescale around 200 ns. Similar positive signal was also observed in the TPV spectrum of pristine BN sample (Figures S13 and S14, Supporting Information), rather suggesting that the electrons were indeed excited from the BN domains. The excited electrons in the pristine BN were then completely quenched without further TPV response as expected. However, the ultrathin structure (≈0.67 nm) of the h‐BN‐C/G‐15 heterojunction significantly extended the lifetime of the excited electrons by significantly shortening the charge transfer path within the exciton Bohr radius (several nanometers). The TPV response of the h‐BN‐C/G‐15 sample is reversed to negative from 5 µs and exhibits a much stronger negative peak at around 240 µs. As depicted in the corresponding scheme of the Figure 4c insets, separated electrons saved in the graphene flowed back to h‐BN until the Fermi level on both sides of the interface becomes the same. The negative peak, the intensity of which is five times of that of positive peak at 200 ns, ends at around 7 ms. The ultralong lifetime of the excited electrons in the atomic‐scale heterojunction matched well with the trend revealed by the fluorescence lifetime results (Figure S15, Supporting Information), due to significantly depressed combination electron–hole pairs during the charge diffusion process and after the charge separation process by the interfacial Schottky barrier. The lowered valance band (Figure S11, Supporting Information) of the h‐BN could principally promote the activation of the substrate molecules, exemplified with benzyl amine here, for further oxidation reactions. In combination of the less amounts of combined electron–hole pairs in the atomic‐scale heterojunction, the optimized h‐BN‐C/G‐15 dyad with obviously enhanced oxidation strength offered the highest turnover frequency (TOF) value (1.58 mmol BA g^−1^ h^−1^) of the h‐BN‐C/G‐15 photocatalyst in this work (Figure 4b), also surpassing the performance of the metal‐free photocatalysts for the same reaction in the literature (Table S6, Supporting Information).

The synergetic effect of the Mott–Schottky photocatalyst on the basis of graphene and h‐BN dyad is general to promote the photocatalytic activation of oxygen molecules and various substituted benzylamines for possible oxidative coupling reactions under visible light irradiation. As shown in Table S5 (Supporting Information), h‐BN‐C/G‐15 photocatalyst gave similar conversions of *ortho*‐, *meta*‐, *para*‐substituted benzylamines with both electron‐withdrawing (e.g., Cl or CF_3_) and electron‐donating (e.g., CH_3_ or OCH_3_) groups. Heterocyclic ring amines with sulfur or nitrogen heteroatom were also converted into the corresponding imines with high selectivity. The excellent and functional‐group tolerance of the Mott–Schottky photocatalyst was again due to its intrinsic activation mechanism by enhancing the electron relocalization of the aromatic rings via the charge‐transfer interaction with conjugated framework of graphene or boron nitride. Note that metal‐based catalysts were usually selective to absorb and activate specific functional groups to accelerate such an oxidative coupling process but were easily poisoned by S or N‐contained functional groups.

In summary, we introduce the construction of Mott–Schottky photocatalysts on the basis of atomic‐scale heterojunctions of boron nitride and graphene (h‐BN‐C/G) for highly efficient artificial photosynthesis. Such an atomic‐scale Mott–Schottky photocatalyst was prepared from the direct copolymerization of glucose, boric acid, and urea with controllable contents of graphene and boron nitride layers. The interfacial Schottky barrier of the ultrathin nanoheterojunction can largely promote the separation of the photogenerated charges carriers for direct activation of the substrate and oxygen molecules. Long‐distance charge transfer path, which could result in obvious combination of separated electrons, was not involved in this atomic‐scale dyadic system. The lifetime of the photogenerated electrons in the h‐BN‐C/G Mott Schottky photocatalysts was thus largely lengthened. All these merits enhanced the photocatalytic activation of oxygen gas for oxidative coupling of amines into imines over the h‐BN‐C/G dyads with high selectivity and reusability under mild conditions.^33^, ^34^ Because of the successful construction of atomic‐scale heterojunction based on graphene and h‐BN monolayers, we expect to synthesize a series of atomic‐scale heterojunctions on the basis of boron, nitrogen, carbon, and other nonmetal atoms^35^, ^36^, ^37^, ^38^, ^39^, ^40^, ^41^, ^42^, ^43^, ^44^ for a wider range of applications.

## Experimental Section

*Materials*: All chemical reagents used in the present experiments were of analytical grade. All the reagents (including urea, glucose, boric acid, acetonitrile, benzylamine, and other derivatives) were purchased from Aladdin Industrial Corporation without purification.

*Preparation of h‐BN‐C/G and Control Samples*: Anhydrous urea and boric acid, with a mass ratio remaining at 40:1, were roughly blended with different amounts of glucose by an agate mortar. After grinding, the mixed precursor was transferred into a covered corundum crucible and heated in a muffle furnace at 1000 °C for 7 h at a heating rate of 2.4 °C min^−1^ under the protection of nitrogen flow. After cooling naturally to room temperature, the as‐obtained samples were directly used for further examination and characterizations. The control sample BN was synthesized without the addition of glucose.

*Oxidative Coupling of Amines into Imines*: 10 mg of the catalysts (h‐BN‐C/G‐15), 5 mL of acetonitrile and benzylamine (0.1 mmol) were added into a 10 mL round‐bottom flask. After the flask was purged with oxygen gas for three times, the mixture was stirred under 1 bar of oxygen at room temperature for 6 h under the visible light irradiation. A 150 W Xe lamp with a water filter was used as the light source for catalytic reactions. The wavelength of the incident light was controlled by using a cutoff filter. The temperature of the reactant solution was maintained at 288 ± 5 K by a water bath during the reaction. The liquid sample for further examination (GC with a flame ionization detector (FID) and/or for GC‐MS analysis) was separated by suction filtration after the reaction.

*Recycling Reaction of Oxidative Coupling of Amines into Imines*: The catalyst was collected by suction filtration and washed with acetonitrile after every reaction was finished. The obtained catalyst was reused for the next catalytic reaction.

*Confirmation of Possible Active Oxygen Species*: Different amounts of scavengers (carotene, 5.37 mg; mannitol, 2 mg; catalase, 35 000 units mL^−1^, 20 µL; BHT, 2.2 mg) were added to identify the active oxygen species on the basis of the standard conditions of oxidative coupling of amines into imines.

*Characterization*: Powder X‐ray diffraction patterns were recorded on a Bruker D8 Advance X‐ray diffractometer with Cu‐Kα radiation (λ = 1.5418 Å with a scan rate of 6° min^−1^. The SEM measurements were performed on a FEI Nova NanoSEM 2300. The microstructure was characterized with a JEM‐2100F TEM (JEOL, Japan) operating at an acceleration voltage of 200 kV. The XPS measurements were conducted on a Kratos Axis Ultra DLD spectrometer using a monochromated Al Kα radiation. Nitrogen sorption experiments (BET) were operated with a Quadrasorb at 77 K, and data analysis was performed with Quantachrome software. Samples were degassed at 150 °C for 16 h before measurements. The AFM measurements were performed on a Multimode NanoscopeIIIa. Raman spectra were acquired using an in Via‐reflexmicro‐Raman spectrometer (Renishaw, UK) with a 532 nm wavelength incident laser. UV–vis absorption spectra were measured on a Hitachi U‐4100 instrument. The GC analysis was performed on Shimadzu GC‐2014gas chromatograph. For TPV measurement, the sample chamber consisted of a glass substrate covered with FTO as top electrode, the same as bottom electrode, and a 10 µm thick mica spacer as electron isolator. The samples were excited with a laser radiation pulse (wavelength of 355 and 532 nm, respectively, and pulse width of 5 ns) from a third harmonic Nd:YAG laser (Polaris, New Wave Research, Inc.). Intensity of the pulse was regulated with a neutral gray filter and determined by an EM500 single‐channel joule meter (Molectron, Inc.). The illuminated area was 0.1 cm^2^. The TPV signals were registered by a 500 MHz digital phosphor oscilloscope (TDS 5054, Tektronix) with a preamplifier.

## Conflict of Interest

The authors declare no conflict of interest.

## Supporting information

Supplementary
