# Supplementary material for: Atomic‐Scale Mott–Schottky Heterojunctions of Boron Nitride Monolayer and Graphene as Metal‐Free Photocatalysts for Artificial Photosynthesis
Source: Adv Sci (Weinh). 2018 May 15;5(7):1800062. doi: 10.1002/advs.201800062 (PMC6051376; doi:10.1002/advs.201800062)
Supplement: Supplementary file 1 — Supplementary [file ADVS-5-1800062-s001.pdf]

Copyright WILEY-VCH Verlag GmbH & Co. KGaA, 69469 Weinheim, Germany, 2018.

ADVANCED SCIENCE Open Access

Supporting Information

for *Adv. Sci.,* DOI: 10.1002/advs.201800062

Atomic-Scale Mott–Schottky Heterojunctions of Boron Nitride Monolayer and Graphene as Metal-Free Photocatalysts for Artificial Photosynthesis

Ke-Xin Zhang, Hui Su, Hong-Hui Wang, Jun-Jun Zhang, Shu-Yu Zhao, Weiwei Lei, Xiao Wei, Xin-Hao Li, and Jie-Sheng Chen**

Supporting Information for

Atomic-scale Mott Schottky Heterojunctions of Boron Nitride Monolayer and Graphene as Metal-free Photocatalysts for Artificial Photosynthesis

Ke-Xin Zhang, Hui Su, Hong-Hui Wang, Jun-Jun Zhang, Shu-Yu Zhao, Weiwei Lei, Xiao Wei, Xin-Hao Li and Jie-Sheng Chen**

K. X. Zhang, H. Su, H. H. Wang, J. J. Zhang, S. Y. Zhao, X. Wei, Prof. X. H. Li* and Prof. J. S. Chen*
School of Chemistry and Chemical Engineering, Shanghai Jiao Tong University, Shanghai 200240, P. R. China.
E-mail: xinhaoli@sjtu.edu.cn (X. H. Li); chemcj@sjtu.edu.cn (J. S. Chen)
Prof. W. Lei
Institute for Frontier Materials, Deakin University, Waurn Ponds Campus, 75 Pigdons Road, Geelong, Victoria 3216, Australia

Figures

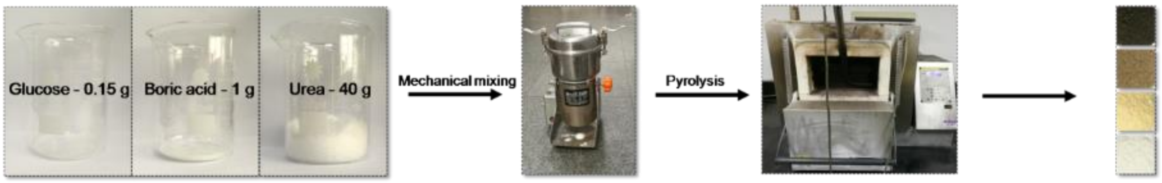

Figure S1. Detailed synthetic process for preparing h-BN-C/G.

a

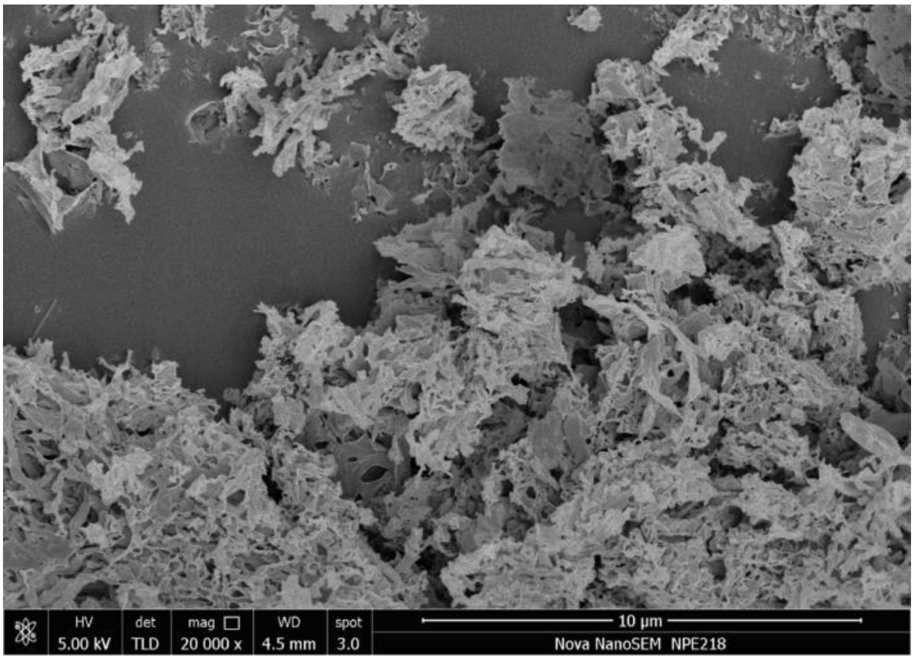

b

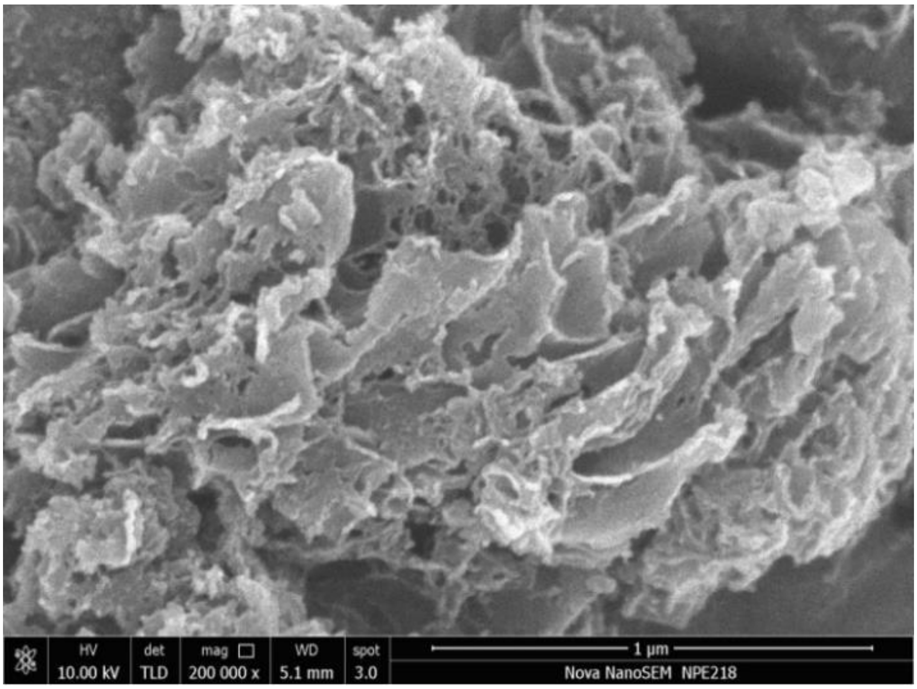

To be continued

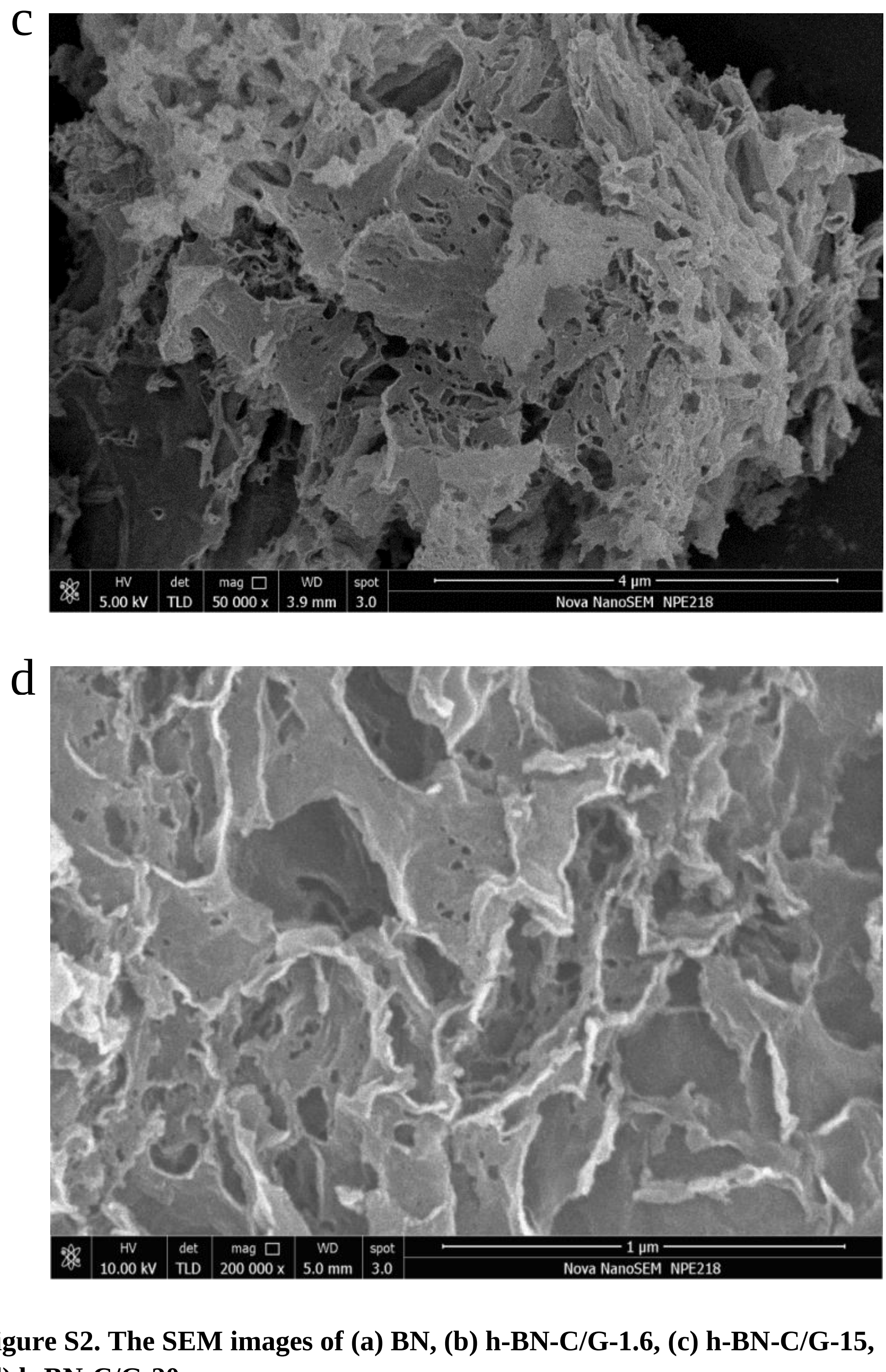

Figure S2. The SEM images of (a) BN, (b) h-BN-C/G-1.6, (c) h-BN-C/G-15, (d) h-BN-C/G-20.

a

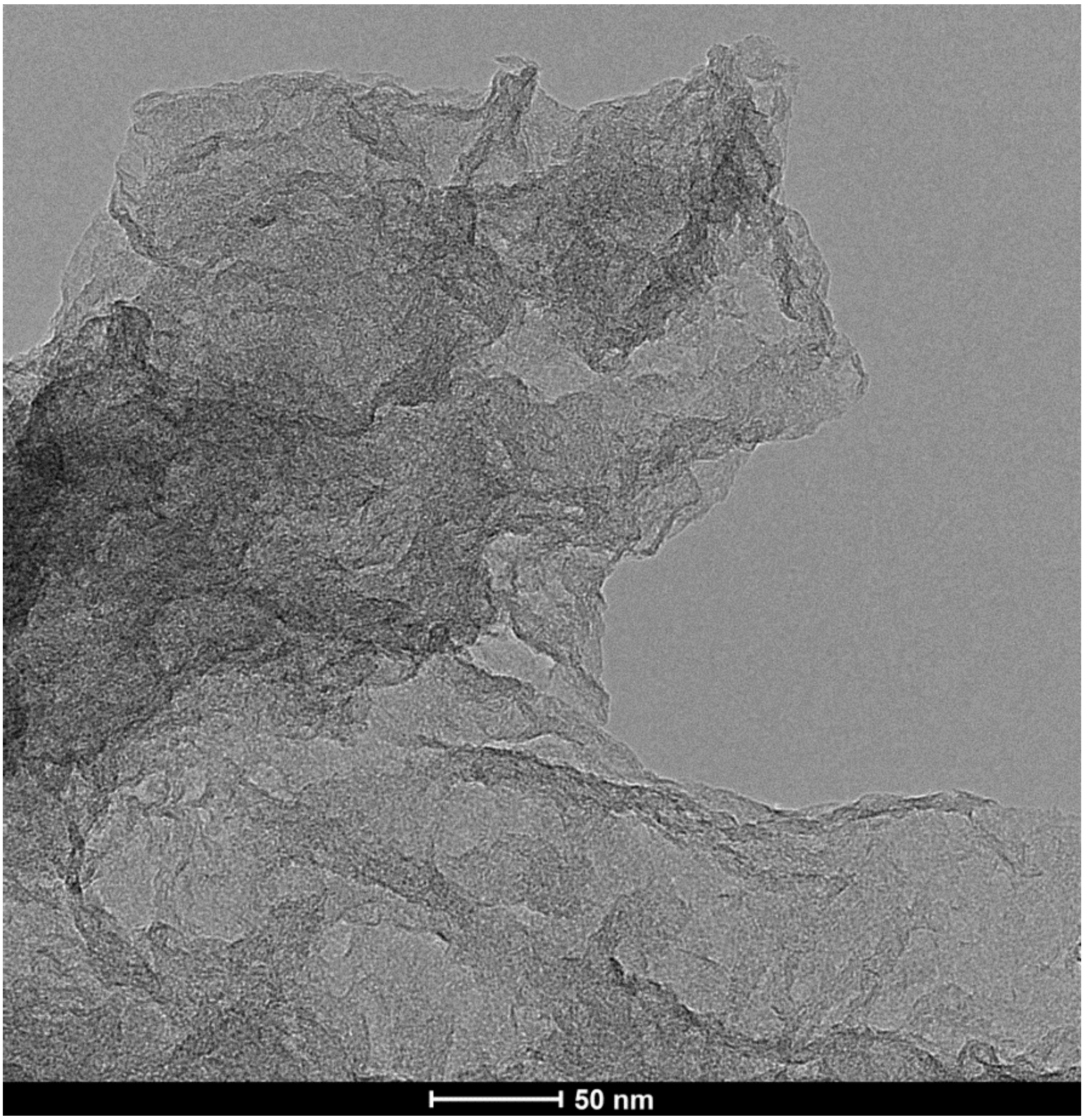

To be continued

b

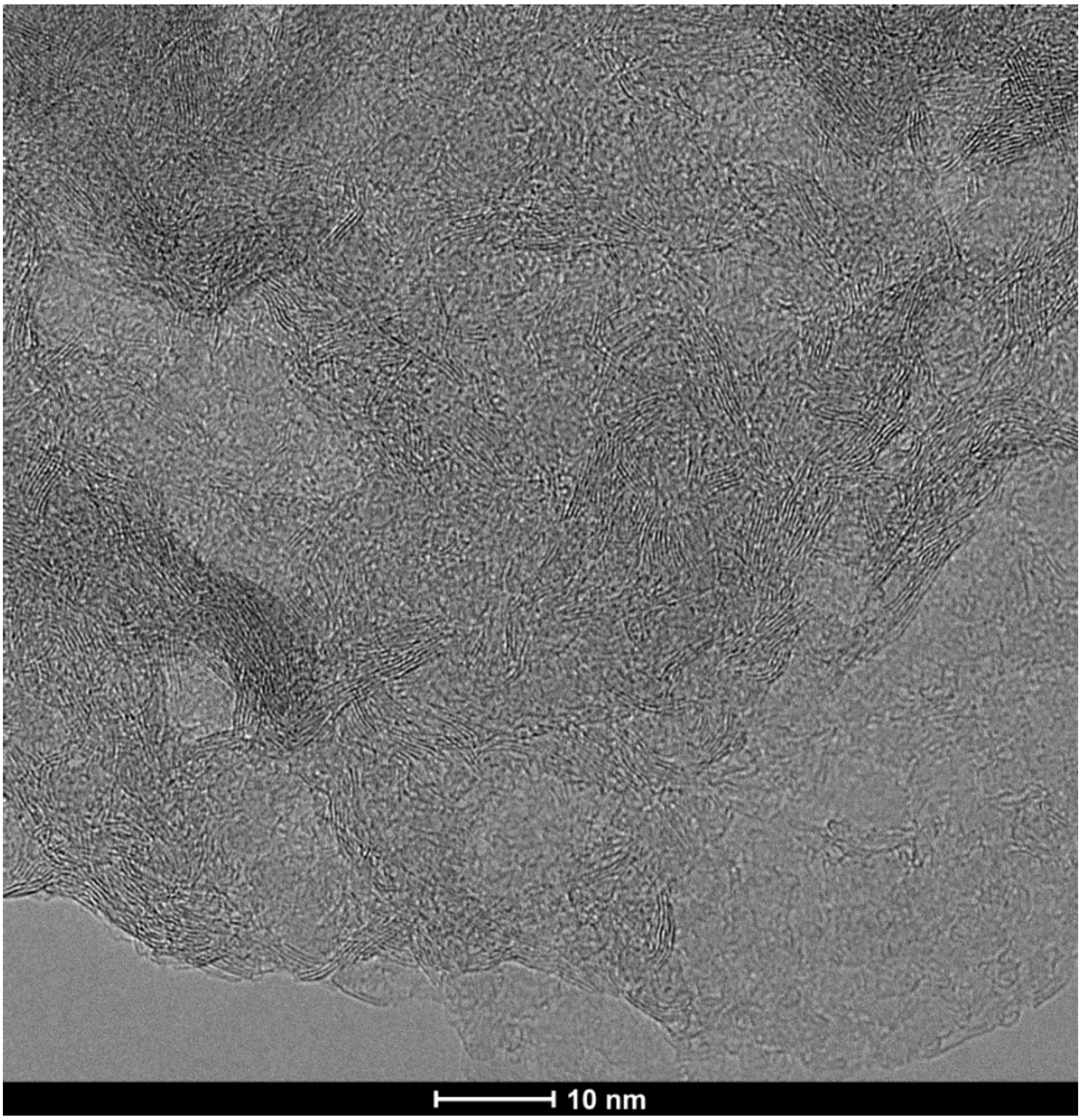

Figure S3. The TEM images of (a, b) BN material.

a

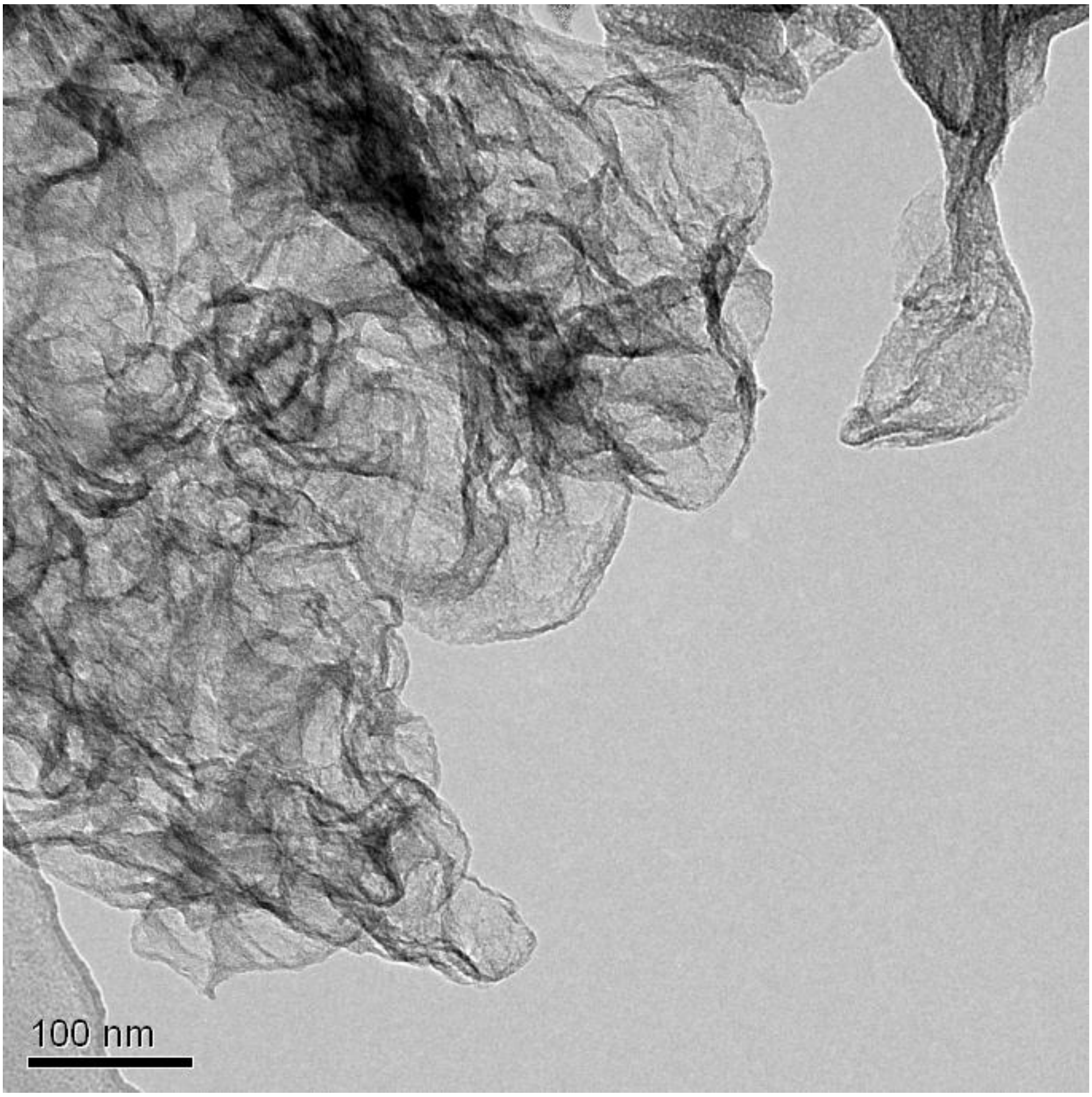

To be continued

b

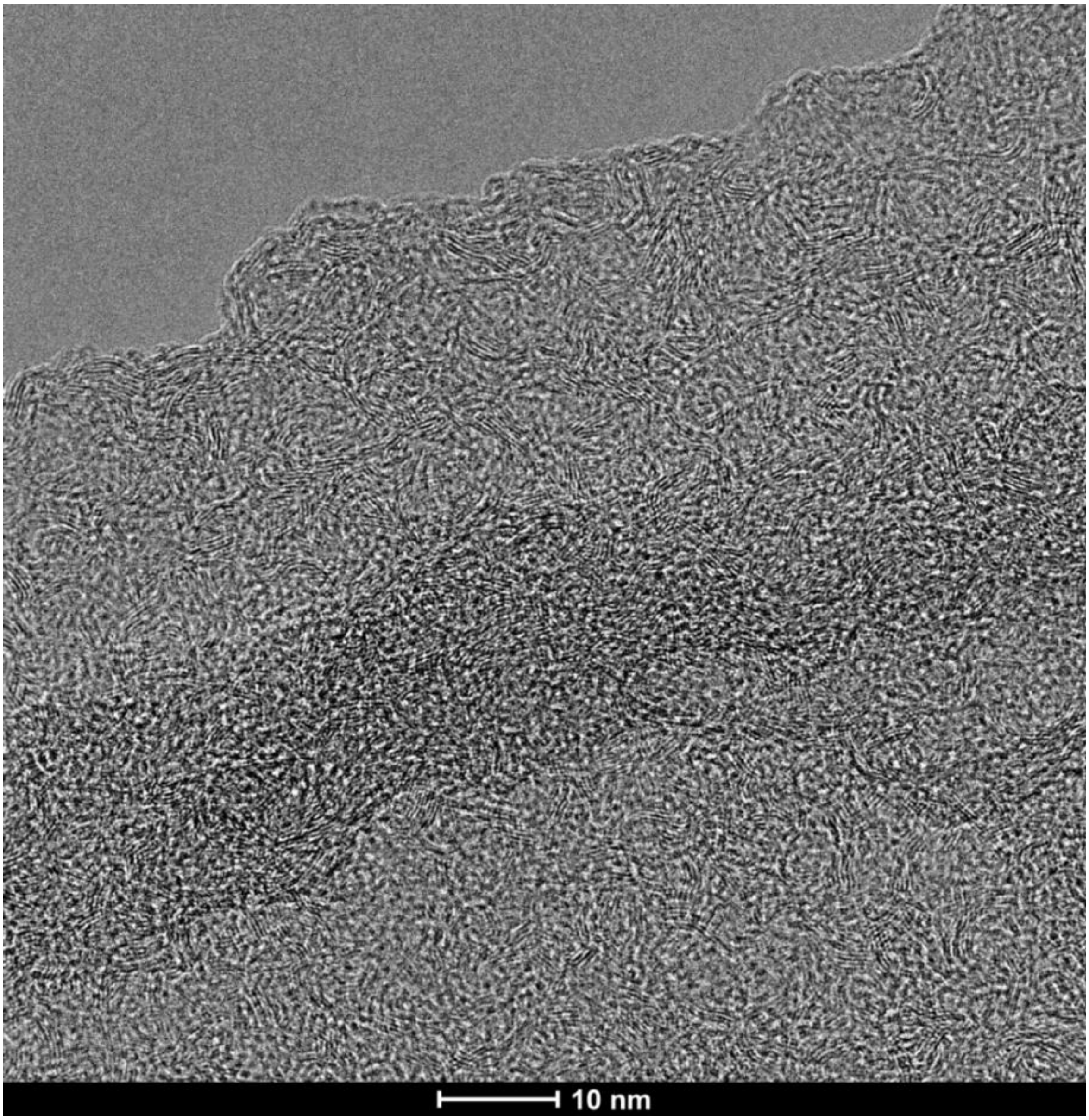

To be continued

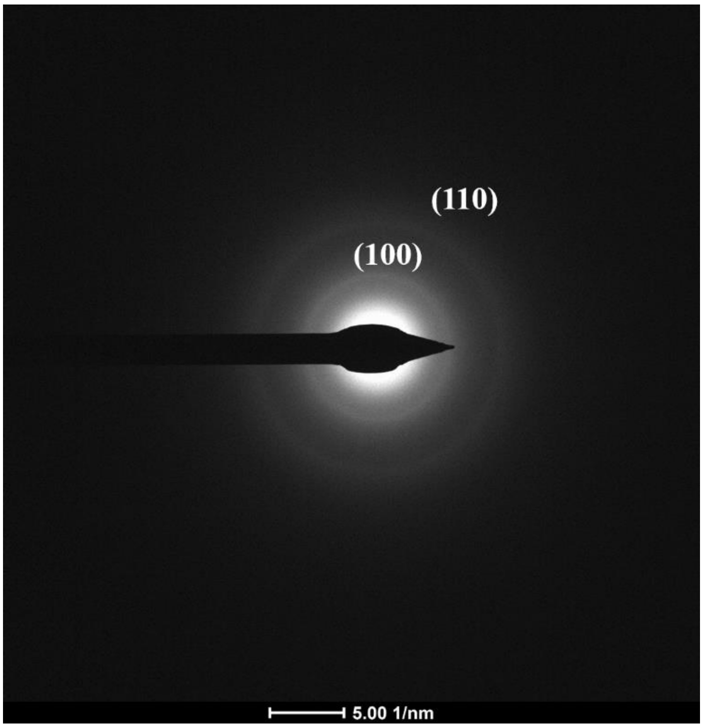

Figure S4. The TEM images of (a, b) h-BN-C/G-15 material; (c) Selected area electron diffraction (SAED) pattern of h-BN-C/G-15 samples. The SAED pattern of the same area presented typical diffraction rings of (110) and (110) facets, consistent to the XRD results. A lattice spacing of 0.22 nm can be assigned to the h-BN (100) planes.

As-produced h-BN-C/G-15 are assemblies of continuous, flexible and wrinkled sheets, similar to patched graphenes.

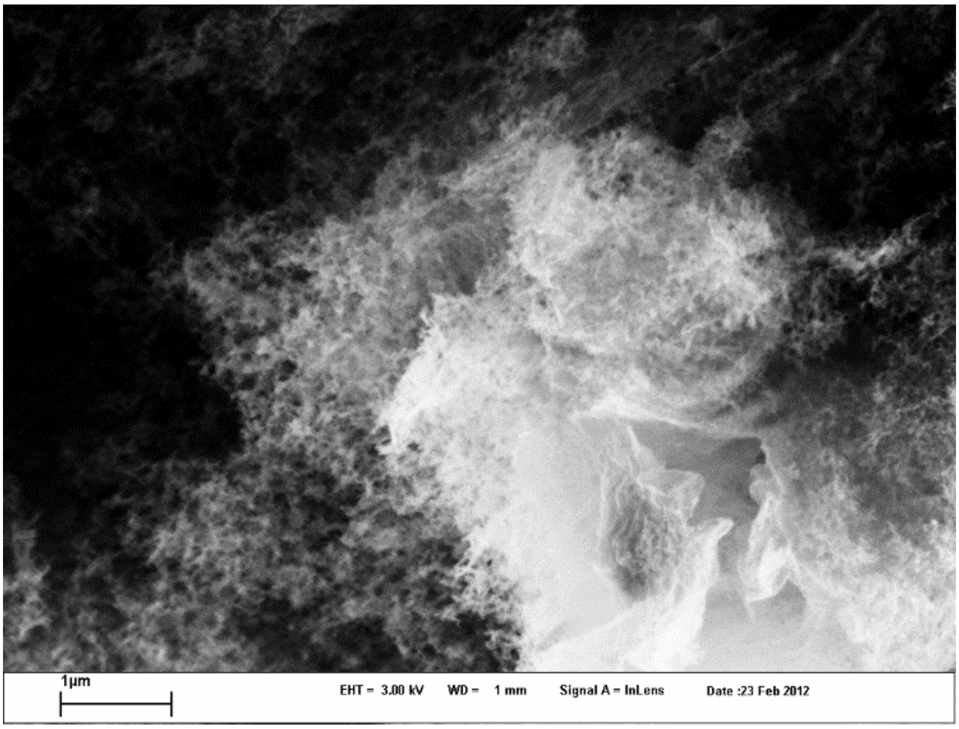

Figure S5. The SEM images of BNHG-1000.

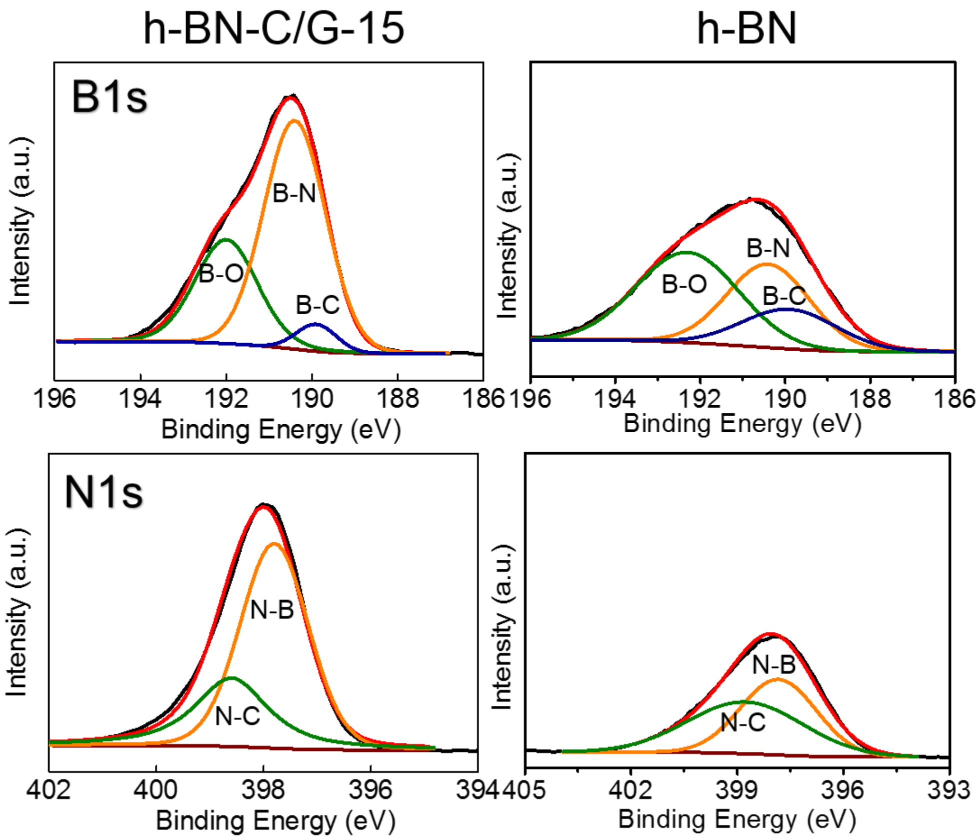

Figure S6. B 1s and N 1s XPS spectra of h-BN-C/G-15 and h-BN. The similar peaks of B-C and N-C band in the B 1s and N 1s XPS spectra of both pristine h-BN and h-BN-C/G-15 samples directly demonstrated the feature of van der Waals heterojunction of boron nitride monolayer and graphene without formation of a significant amount of chemical bonds between them.

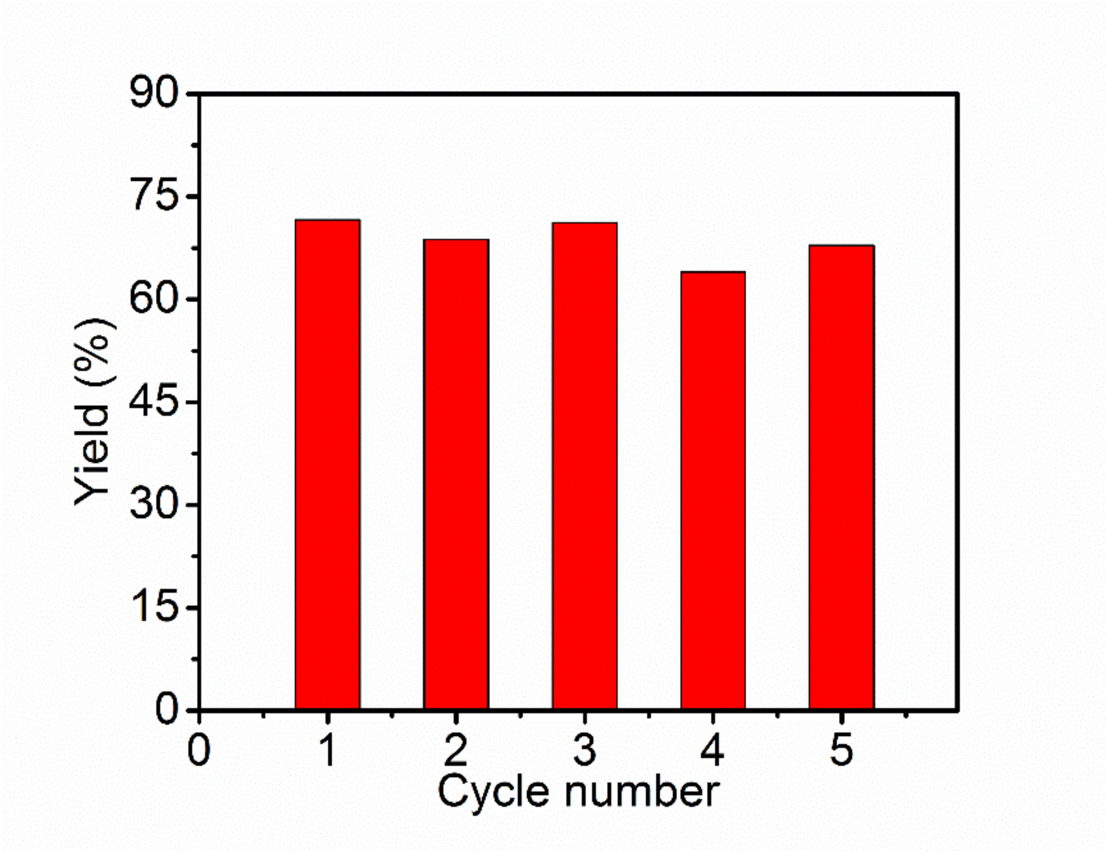

Figure S7. Cycling reactions over h-BN-C/G-15 for 4 hours using oxygen gas and visible light (λ > 420 nm) at room temperature.

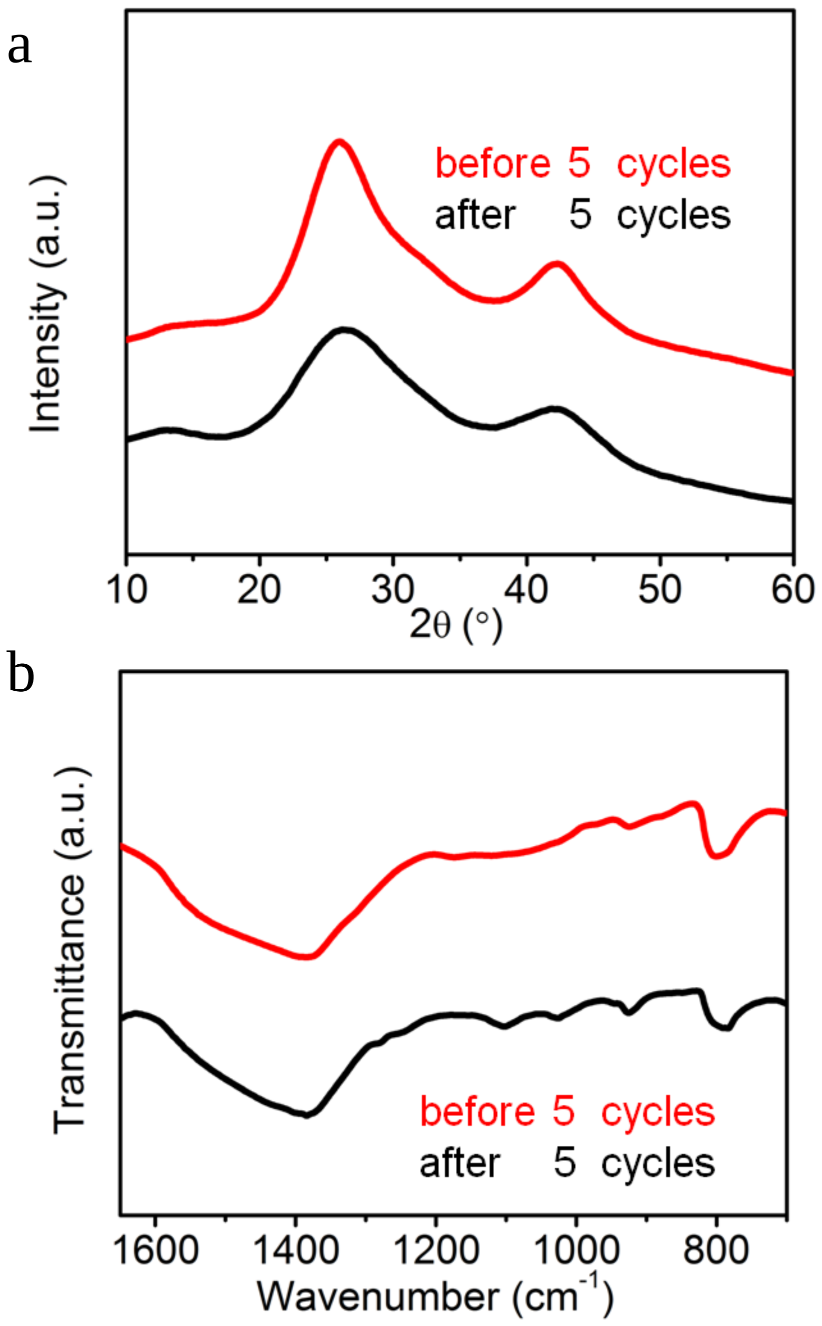

Figure S8. (a)XRD and (b) FTIR spectra of the h-BN-C/G-15 samples before and after 5 cycles respectively. The high yields and changeless structure revealed the excellent stability of h-BN-C/G-x samples.

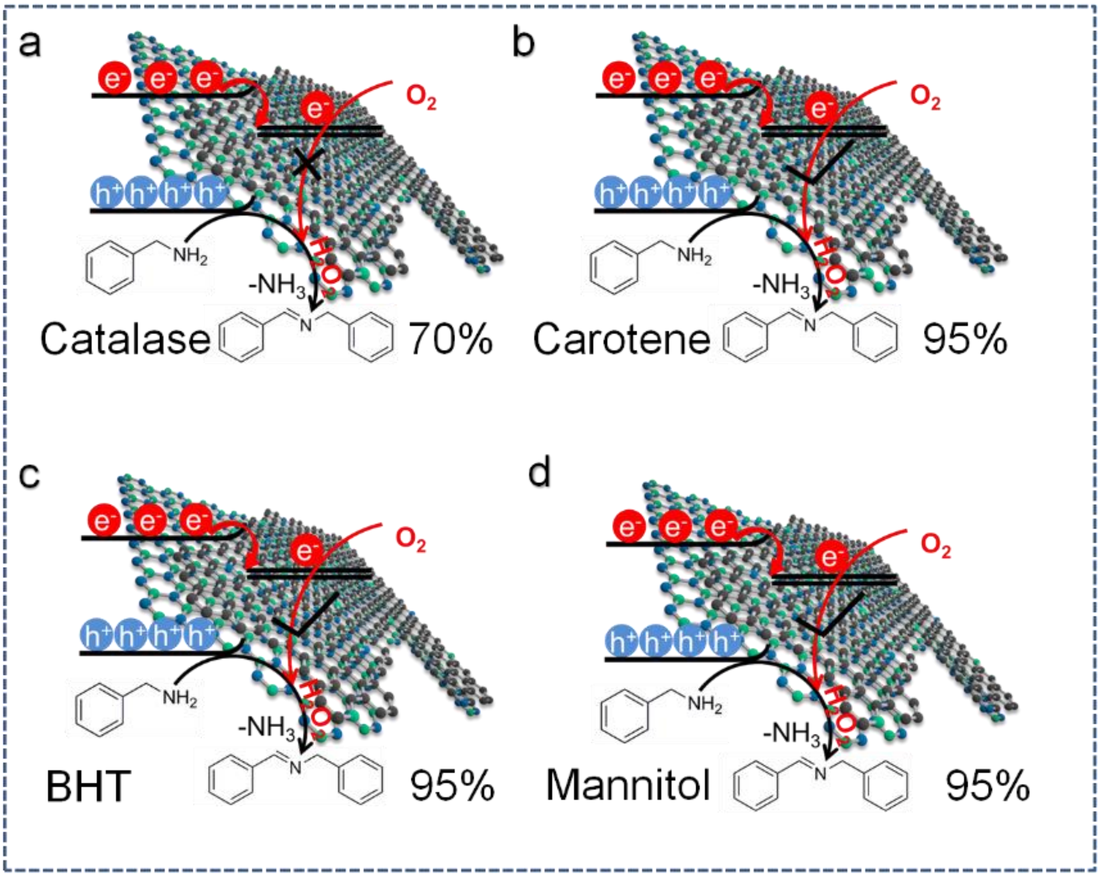

Figure S9. Different reaction conversion of benzylamine into imine over h-BN-C/G-15 in the presence of (a) catalase, (b) carotene, (c) BHT, (d) mannitol. After adding catalase into the reaction system, the conversion of the reaction is obviously decreased, which reveals the formation of H_2O_2 as the generated reactive oxygen species here.

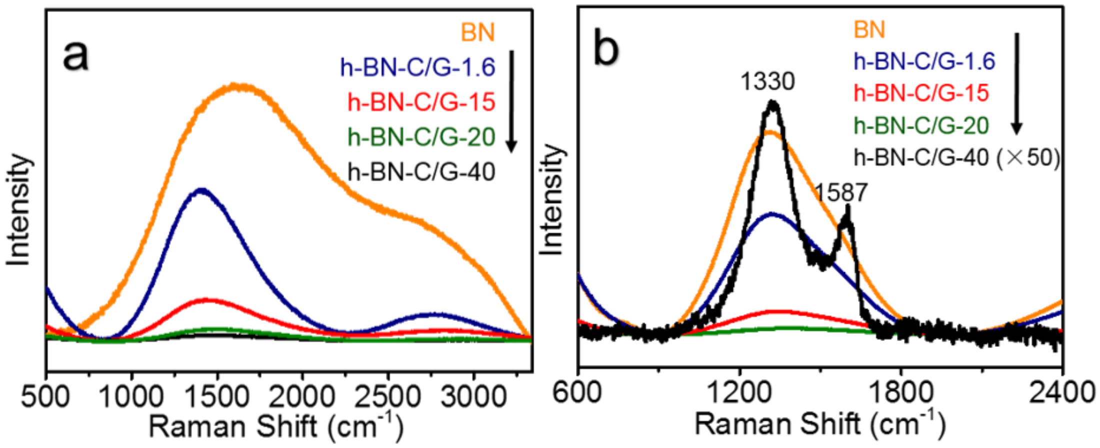

Figure S10. **Raman spectra of BN, h-BN-C/G-x at (a) 532 nm and (b) 780 nm of excitation lase wavelength.**

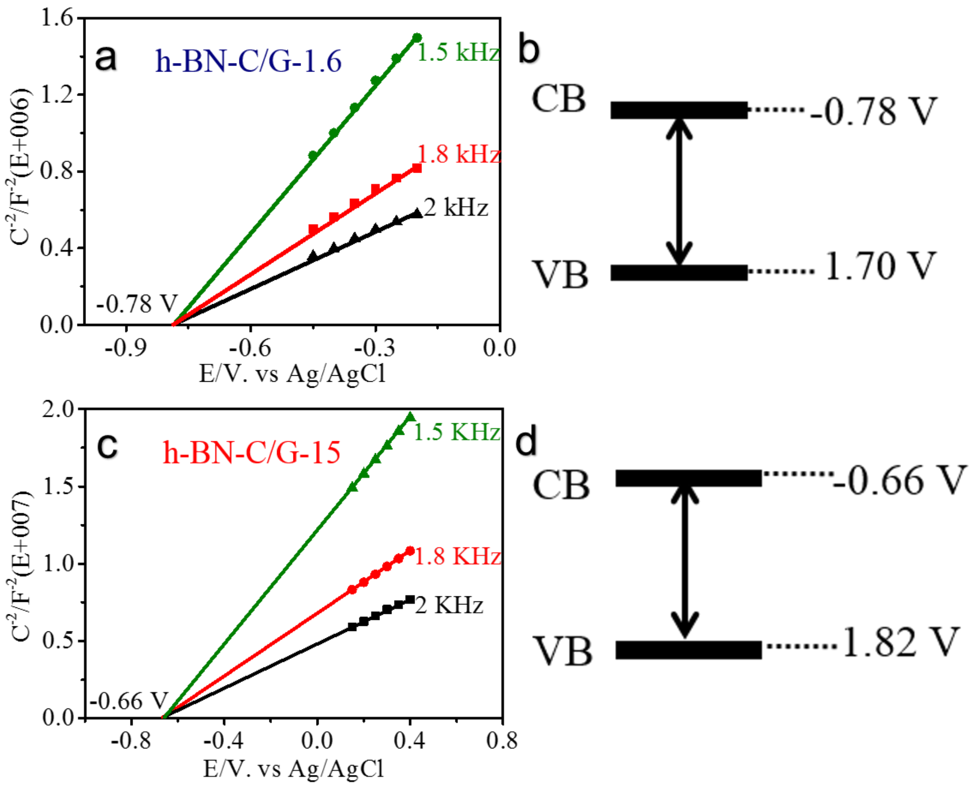

Figure S11. (a)Mott–Schottky plots of h-BN-C/G-1.6 at selected frequencies of 1.5 kHz, 1.8 kHz and 2 kHz; (b) The LUMO (CB) and HOMO (VB) for h-BN-C/G-1.6 on a potential scale (V) versus Ag/AgCl. CB, conduction band; VB, valence band; (c) Mott–Schottky plots of h-BN-C/G-15 at selected frequencies of 1.5 kHz, 1.8 kHz and 2 kHz; (d) The LUMO (CB) and HOMO (VB) for h-BN-C/G-15 on a potential scale (V) versus Ag/AgCl. CB, conduction band; VB, valence band.

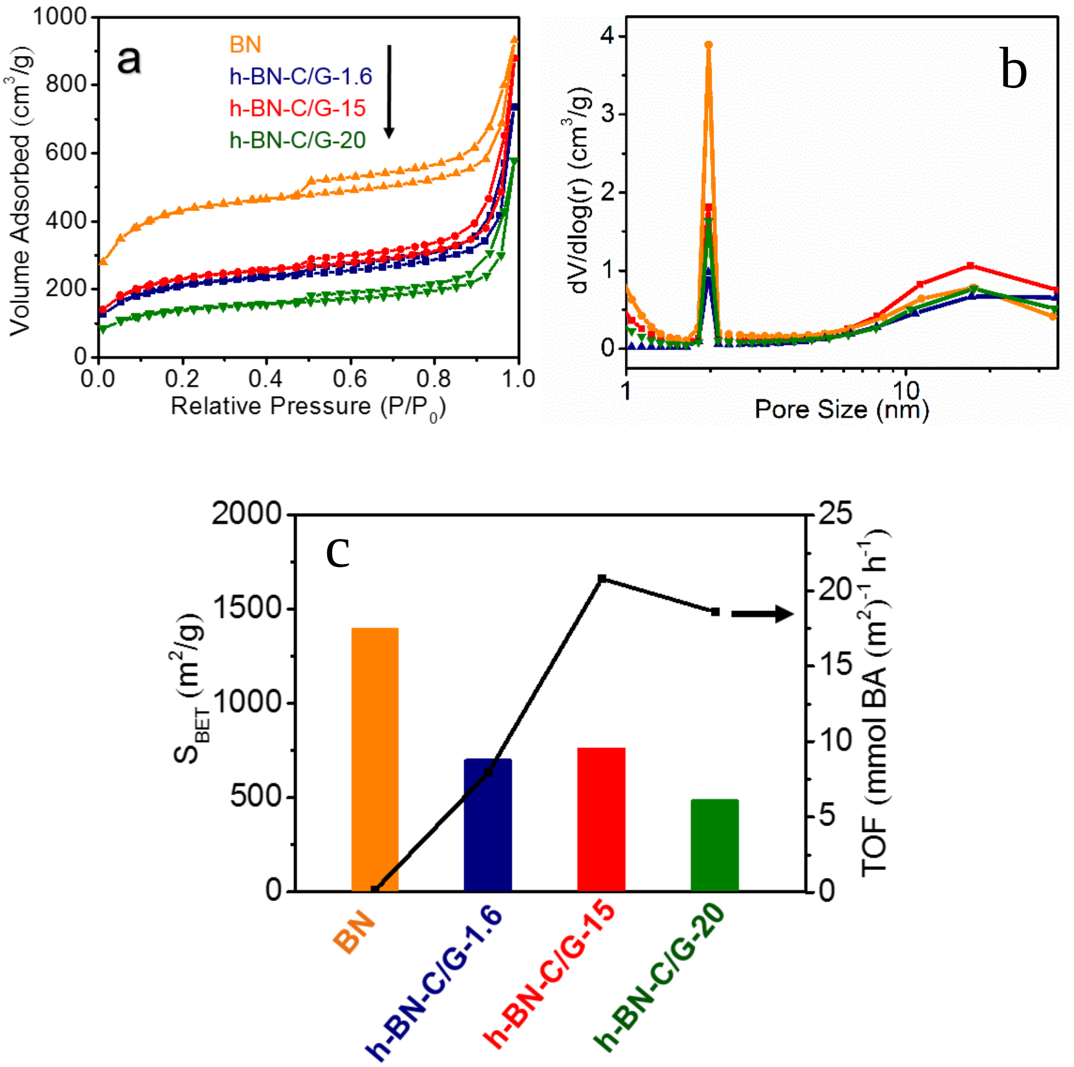

Figure S12. (a) N_2 absorption-desorption plots, and (b) pore-size distribution of h-BN-C/G-x samples, (c) the normalized TOF values *versus* surface area of h-BN-C/G-x samples. It was noted that gradual decrease in the BET surface areas of BN, h-BN-C/G-1.6, h-BN-C/G-15, h-BN-C/G-20 samples was mainly induced by the formation of multilayer heterojunctions. The normalized TOF values *versus* surface area also indicated an unchanged trend of the varied activities of all h-BN-C/G-x samples, rather speaking for a minor contribution of the surface area to the final catalytic performance.

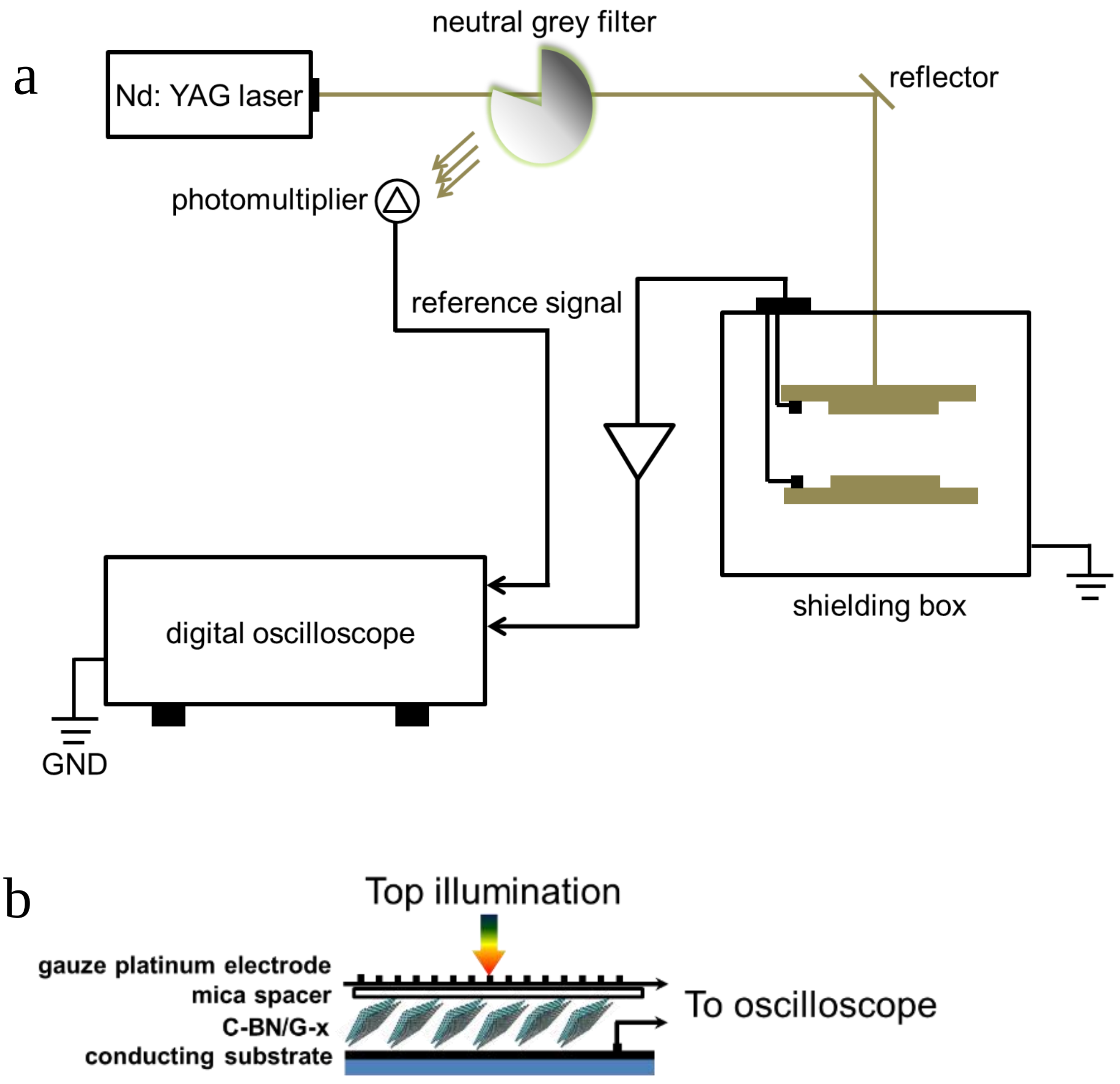

Figure S13. (a) Schematic diagram of Transient photovoltage (TPV) experimental set-up, and (b) details of sample chamber in the shielding box (the conducting substrate is FTO).

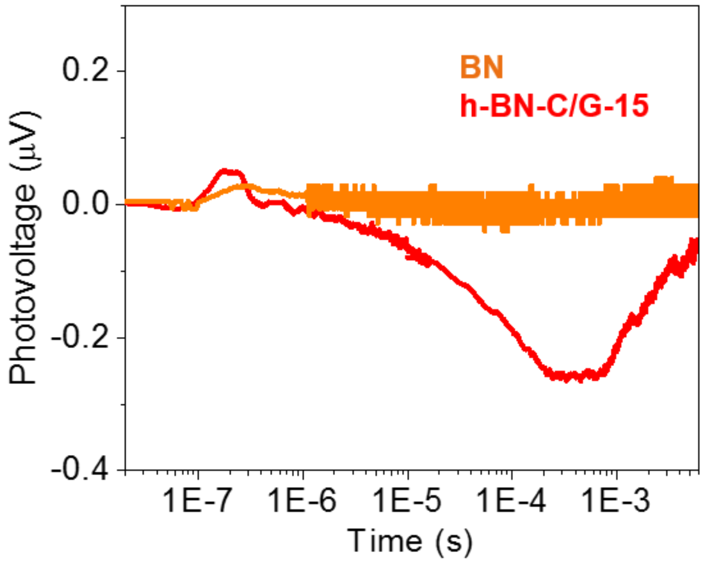

Figure S14. Transient photovoltage (TPV) spectrum of BN and h-BN-C/G-15 samples.

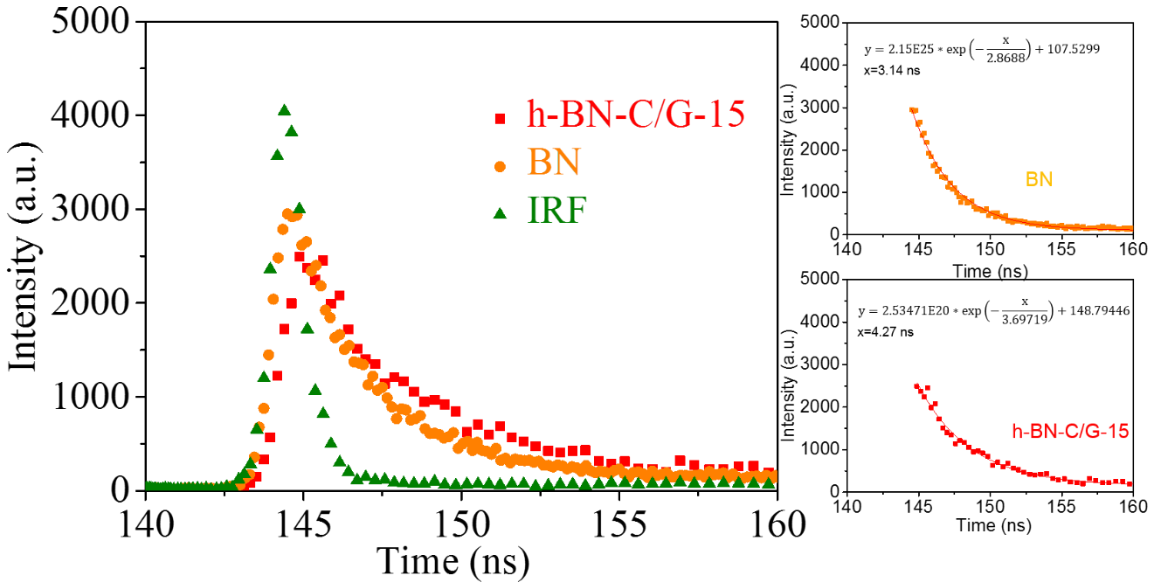

Figure S15. Time-resolved fluorescence spectra of BN and h-BN-C/G-15. According to the photovoltage transient (TPV) data (Figure 4c and Figure S14), we could find that the positive signals of h-BN-C/G-15 and pristine h-BN sample are similar and comparable, whilst a reversed TPV to negative was only observed in the hybrid sample (h-BN-C/G-15).The ultra-lone lifetime of the excited electrons in the atomic-scale heterojunction matched well with the trend revealed by the fluorescence lifetime results, all due to significantly depressed combination electron-hole pairs during the charge diffusion process and after the charge separation process by the interfacial Schottky barrier.

Tables

Table S1. Compositions of various h-BN-C/G-x materials.

Catalysts	C (at. %)	B (at. %)	N (at. %)	O (at. %)
BN	11.53	32.47	29.83	21.92
h-BN-C/G-1.6	12.15	32.25	27.95	23.20
h-BN-C/G-15	20.02	30.20	29.84	21.18
h-BN-C/G-20	40.75	18.08	17.31	21.65

Table S2. Photocatalytic oxidation of benzylamine over h-BN-C/G-15 in different solvents.

Entry	Solvent	Conversion [%]	Selectivity [%]
1	acetonitrile	95	>99
2	water	8.71	>99
3	methylbenzene	66.7	>99
4	DMF	>99	87.9
5	ethanol	71.1	78.2

As illustrated in Table S2, the reaction conversion is highest in the DMF solvent among the five solvents. The reaction selectivity is supreme in the acetonitrile, water, and methylbenzene among the five solvents. In general, choosing acetonitrile as reaction solvent is sensible in this reaction.

Table S3 The activity of h-BN-C/G-x for the oxidative coupling reactions of amines[a].

Entry	Catalyst	Conversion[%]	Selectivity[%]
1	BN	trace	>99
2	h-BN-C/G-1.6	33.5	>99
3	h-BN-C/G-5	53.5	>99
4	h-BN-C/G-10	83.6	>99
5	h-BN-C/G-15	95.0	>99
6	h-BN-C/G-20	54.2	>99

Reaction conditions: [a] Benzylamine (0.1 mmol), catalyst (10 mg), acetonitrile (5 ml), O_2 balloon (1 bar), light source: $\lambda > 420$ nm, reaction time: 6h, room temperature.

Table S4. The S_{BET} and reaction conversion of h-BN-C/G-x materials.

Entry	Catalyst	Conversion [%]	S_{BET} (m^2/g)
1	BN	1.27	1394.62
2	h-BN-C/G-1.6	33.5	700.21
3	h-BN-C/G-15	95.0	761.91
4	h-BN-C/G-20	54.2	484.6

Table S5. Photocatalytic oxidation of various amines to imines with h-BN-C/G-15 as catalyst.[a]

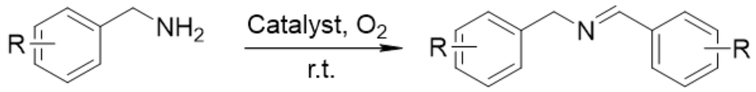

Entry	Substrate	Conversion[%]	Selectivity[%]
1	R=H	95.0	>99
2	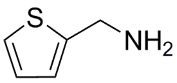	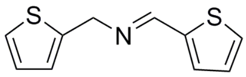 95.0	>99
3	R=2-CH_3	70.3	>99
4	R=3-CH_3	82.6	>99
5	R=4-CH_3	82.4	>99
6	R=2-OCH_3	80.7	>99
7	R=3-OCH_3	94.5	>99
8	R_1=2-OCH_3 R_2=5-OCH_3	82.6	>99
9	R=4-CF_3	82.1	>99
10	R=4-Cl	75.5	>99
11	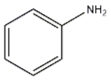	-	-

[a] Benzylamine (0.1 mmol), catalyst (10 mg), acetonitrile (5 mL), O_2 balloon (1 bar), light source: λ >420 nm, reaction time: t = 6h, room temperature.

Table S6. The performance of the metal-free photocatalysts for the oxidative coupling reactions of amines to imines in the literatures.

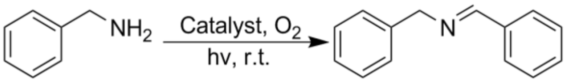

Catalyst	Pressure	T	λ (nm)	TOF [mmol BA g^{-1} h^{-1}]	Ref.
h-BN-C/G-15	1 atm	r.t.	>420	4.0	This work
m-O=C_3N_4-2.3	1 atm	r.t.	>420	1.14	[1]
mpg-C_3N_4	0.5 Mpa	303 K	>420	1.3	[2]
Phenothiazine Dyes	1 atm	r.t.	Blue LED	0.11	[3]

Reference

[1] J. J. Zhang, J. M. Ge, H. H. Wang, X. Wei, X. H. Li, J. S. Chen, *ChemCatChem.* **2016**, 8, 3441-3445.

[2] F. Su, S. C. Mathew, L. Möhlmann, M. Antonietti, X. Wang, S. Blechert, *Angew. Chem. Int. Ed.* **2011**, 50, 657-660.

[3] J. H. Park, K. C. Ko, E. Kim, N. Park, J. H. Ko, H. Ryu, T. K. Ahn, J. Y. Lee, S. U. Son, *Org. Lett.* **2012**, 14, 5502.
